# Emergence of Barmah Forest virus in Western Australia.

**DOI:** 10.3201/eid0101.950104

**Published:** 1995

**Authors:** M Lindsay, C Johansen, A K Broom, D W Smith, J S Mackenzie

**Affiliations:** Department of Microbiology, University of Western Australia, Queen Elizabeth II Medical Centre, Nedlands, WA, Australia.


### Emergence of Barmah Forest Virus in Western Australia[1]

**To the editor:** Barmah Forest (BF) virus is a mosquito-borne alphavirus, found only in Australia, which causes outbreaks of polyarthritis in humans. The disease is very similar to epidemic polyarthritis caused by infection with Ross River virus, another Australian alphavirus. BF virus was first isolated from mosquitoes in the State of Western Australia in 1989. After this, small clusters of human cases were diagnosed in the arid northern and central regions of Western Australia in 1992, and the first substantial outbreak of human disease due to infection with BF virus (BF virus disease) occurred in the southwestern region of the state during the spring and summer (September-March) of 1993-94 (2). No evidence of BF virus activity had been found in these regions before these events, which suggests that the virus had only recently been introduced to Western Australia. This report describes the timing and distribution of BF virus disease in humans and the isolation of the virus from mosquitoes in Western Australia, which corroborate the view that BF virus is an emerging virus in this state.

The ecology of Australian arboviruses that cause human disease, including BF virus, has recently been reviewed (3). BF virus was first isolated from *Culex annulirostris* mosquitoes collected at the Barmah Forest in northern Victoria (southeastern Australia) in 1974 (4). It was first shown to infect humans in New South Wales (central-eastern and southeastern Australia) in 1986 (5) and was reported as a cause of clinical disease in humans in 1988 (6). The most common clinical features include polyarthritis, arthralgia, myalgia, fever, rash, and lethargy (7); in some cases, symptoms may persist for more than 6 months (2). Although the symptoms are similar to those caused by infection with Ross River virus, there is little cross-reaction between the two viruses in serologic tests (8), and differentiating between infections caused by either is generally not difficult. The first true outbreak of BF virus disease occurred concurrently with an outbreak of Ross River virus infection at Nhulunbuy in the Northern Territory in early 1992 (9).

The principal vectors of BF are believed to be mosquitoes, and although the vertebrate hosts of BF virus are not known, serologic surveys in eastern Australia have suggested that marsupials are involved in the natural cycle.

BF virus was first detected in Western Australia in 1989. Since then, 73 isolates of the virus have been obtained from mosquitoes trapped in several different regions of Western Australia (Table 1). The first human cases of BF virus disease in Western Australia were reported in 1992, and 67 serologically confirmed cases have now been diagnosed. The locations of towns where human cases have occurred or where mosquitoes that yielded BF virus were collected are shown in Figure 1.

Eight isolates of the virus were obtained from five different mosquito species (Table 1) collected at Billiluna, a small, remote aboriginal community in an arid area in the southeastern Kimberley region in April 1989 (10). The infected mosquitoes were collected 3 weeks after heavy local rains. Only moderate wet season rains were recorded in the remainder of the Kimberley region, and no cases of BF virus disease were reported from any region in Western Australia that year. There have been no subsequent isolations of BF virus from mosquitoes collected at Billiluna, despite annual collections in the region. No human cases have been reported from Billiluna.

The first cases of BF virus disease in Western Australian were reported almost 3 years later, either individually or in small clusters from towns in the arid East Kimberley, Pilbara, Gascoyne, Murchison and Southeast (Goldfields) regions between April and September (Autumn-Spring) 1992. Most activity was reported from the towns of Exmouth (six cases) and Carnarvon (four cases). All of these cases occurred during or just after much larger outbreaks of disease caused by Ross River virus. This suggested that BF and Ross River viruses may have similar mosquito vectors and require similar environmental conditions for successful transmission. The main environmental factor contributing to the 1992 outbreaks of Ross River virus disease was extremely heavy rain in these normally arid regions during autumn and winter (11).

BF virus was isolated from five species of mosquito in the Fortescue region of the Pilbara and from three species in the West Gascoyne, just prior to, and during, these arid-region outbreaks. In coastal regions of the Pilbara, the main vector of BF virus appears to be *Aedes vigilax*, a salt marsh-breeding species. Large numbers of this species develop after very high tides or heavy rains on salt marshes. It is also the main vector of Ross River virus in these regions (12). Several other temporary freshwater ground pool-breeding species in the subgenus *Ochlerotatus*, particularly *Ae. eidsvoldensis* and *Ae.* EN Marks' species #85, were found to be infected with the virus in inland areas or coastal areas where such pools develop. These preliminary investiga-

[1] This report is adapted from and expands on a previous bulletin. (1)

**Table 1. Mosquito species from which BF virus was isolated in Western Australia by region and date, 1989–1993***

| Region† | Locality† | Species | Date | Isolates |
|---|---|---|---|---|
| East Kimberley | Billiluna | *Ae. bancroftianus* | 22 Apr 1989 | 1 |
| East Kimberley | Billiluna | *Ae. eidsvoldensis* | 22 Apr 1989 | 3 |
| East Kimberley | Billiluna | *Ae. pseudonormanensis* | 22 Apr 1989 | 1 |
| East Kimberley | Billiluna | *An. amictus* | 22 Apr 1989 | 2 |
| East Kimberley | Billiluna | *An. annulipes* s.l. | 22 Apr 1989 | 1 |
| East Kimberley | Halls Creek | *Cx. annulirostris* | 11 Feb 1993 | 1 |
| West Kimberley | Broome | *Ae. vigilax* | 10-16 Feb 1993 | 9 |
| West Kimberley | Fitzroy Crossing | *Cx. annulirostris* | 13 Feb 1993 | 1 |
| West Kimberley | Willare | *Ae. normanensis* | 16 Mar 1993 | 1 |
| Pilbara (Fortescue) | Onslow | *Ae.* EN Marks' sp. #85 | 13-14 Jun 1992 | 3 |
| Pilbara (Fortescue) | Onslow | *Cx. annulirostris* | 13 Jun 1992 | 1 |
| Pilbara (Fortescue) | Onslow | *An. amictus* | 13-14 Jun 1992 | 2 |
| Pilbara (Fortescue) | Exmouth | *Ae. vigilax* | 16 Jun-11 Jul 1992 | 7 |
| Gascoyne (West) | Minilya | *Ae. eidsvoldensis* | 7 Jul 1992 | 5 |
| Gascoyne (West) | Minilya | *Ae. eidsvoldensis* (bloodfed) | 7 Jul 1992 | 1 |
| Gascoyne (West) | Minilya | *Ae.* EN Marks' sp. #85 | 7 Jul 1992 | 1 |
| Gascoyne (West) | Carnarvon | *Ae. eidsvoldensis* | 12 Jul 1992 | 3 |
| Gascoyne (West) | Carnarvon | *Ae.* EN Marks' sp. #85 | 12 Jul 1992 | 1 |
| Gascoyne (West) | Carnarvon | *Cx. quinquefasciatus* | 12 Jul 1992 | 1 |
| Gascoyne (West) | Carnarvon | Unidentifiable mosquitoes | 12 Jul 1992 | 1 |
| Central Coastal | Karnup | *Cx. annulirostris* | 4 Jan 1993 | 1 |
| Central Coastal | Karnup | *Cq.* species near *linealis* | 4 Jan 1993 | 4 |
| Central Coastal | Perth | *Cx. annulirostris* | 6 Jan 1993 | 1 |
| South Coastal | Australind | *Ae. camptorhynchus* | 6 Jul 1993 | 1 |
| South Coastal | Mandurah (Peel) | *Ae. camptorhynchus* | 5 Aug-5 Oct 1993 | 10 |
| South Coastal | Busselton | *Ae. camptorhynchus* | 1 Sep-15 Nov 1993 | 9 |
| South Coastal | Busselton | *Cx. globocoxitus* | 1 Nov 1993 | 1 |
| | | | | **Total 73** |

* Numbers of mosquitoes trapped and processed and estimated minimum field infection rates for each region will be published in detail elsewhere.
† Refer to Figure 1 for location of regions and towns from which isolates of BF virus were obtained.

tions also suggested that both BF and Ross River virus can co-circulate. Both viruses were isolated from different mosquitoes of the same species collected in the same trap on several occasions.

A further six cases of BF virus disease were reported after record wet season rains in the Kimberley region in early 1993. The cases occurred just after mosquitoes in the Kimberley region had been collected by personnel from this laboratory. These collections yielded 12 isolates of BF virus. Eleven of these were from *Ae. vigilax* and *Cx. annulirostris* mosquitoes trapped less than 2 weeks after the first heavy wet season rains near the West Kimberley towns of Broome and Fitzroy Crossing and the East Kimberley town of Halls Creek (see Figure 1 for locations, Table 1 for isolation details). A twelfth isolate was obtained from *Ae. normanensis* collected 5 weeks after the first rains at Willare in the West Kimberley. The timing of the collections was such that all three mosquito species could have transmitted BF virus to the infected persons in the region. Vector competence studies are required to determine if one or more species were likely to have been the main vectors.

A single case was reported from the metropolitan area of Perth, the state's capital, in August 1992. This was the first evidence of BF virus activity in the temperate and populous southwestern region of Western Australia. However, the travel history of the patient was not obtained. Then, in early January

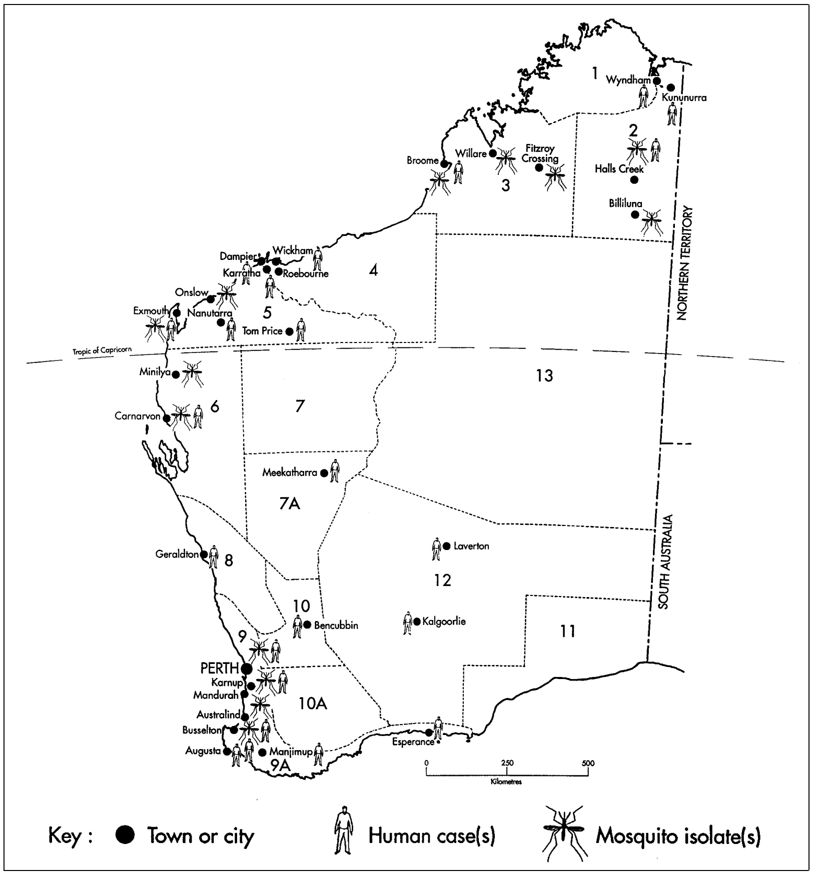

Figure 1. Meteorological regions and towns in Western Australia where human cases of Barmah Forest virus disease were reported and Barmah Forest virus was isolated from mosquitoes, 1989–1994. Meteorologic regions: 1. North Kimberley; 2. East Kimberley; 3. West Kimberley; 4. Pilbara (De Grey); 5. Pilbara (Fortescue); 6. West Gascoyne; 7. East Gascoyne; 7A. Murchison; 8. North Coastal; 9. Central Coastal; 9A. South Coastal; 10. North Central; 10A. South Central; 11. Eucla; 12. Southeast (Goldfields); and 13. Northeast (Interior).

1993, this laboratory isolated BF virus from *Cx. annulirostris* and *Coquillettidia* species near *linealis*[2] mosquitoes collected at Karnup, south of Perth (Table 1). A week later a single human case was reported from an address near the site at which the mosquitoes were trapped. BF virus was also isolated from *Cx. annulirostris* mosquitoes trapped in the southern suburbs of Perth in early January 1993, providing evidence that the virus may be transmitted to humans in the metropolitan area. Two further cases were reported from the Perth metropolitan area, one in February and one in May 1993. Again, no travel histories were available for these patients, but it appears that the virus remained and was actively transmitted in the southwest during the autumn and winter of 1993, as it was isolated from *Ae. camptorhynchus* mosquitoes trapped in July and August (Table 1).

A larger outbreak of BF virus disease occurred in the southwest region between September 1993 and March 1994. This has been described in detail elsewhere (2). Twenty-eight serologically confirmed cases were reported from the southwest region during that period. Of these, more than half (17 cases) were in or near the small coastal towns of Mandurah (Peel) and Busselton during spring (September-November) of 1993. BF virus was isolated on 20 occasions from pools of *Ae. camptorhynchus* mosquitoes collected in the Mandurah and Busselton regions before and during the outbreak (Table 1), thereby implicating that species, along with *Cx. annulirostris* and *Cq.* species near *linealis,* as an important vector in the southwest. *Ae. camptorhynchus* breeds in salt marshes and brackish wetlands during all but the hottest months of the year in the southwest and is the main vector of Ross River virus in the region (3, 11). The ratio of the carriage rate of BF virus in *Ae. camptorhynchus* during the outbreak to the number of human cases was very high compared with the rate observed for Ross River virus in *Ae. camptorhynchus* in the same regions during previous Ross River virus outbreaks (M.D. Lindsay, C.A. Johansen, and J.S. Mackenzie, unpublished observations). This suggests that the ratio of subclinical (therefore unreported) to clinical cases may be much larger with BF virus than with Ross River virus or that fewer humans were infected, possibly because *Ae. camptorhynchus* may not transmit BF virus to humans as efficiently as it does Ross River virus. Seven cases were reported between November 1993 and March 1994 in small towns in the inland southwest region in a later cycle of virus activity. Unfortunately, no collections of mosquitoes were carried out in the region during that time. Small clusters of cases or individual cases were also reported from several other regions of Western Australia during this time, including three additional cases from Broome in the West Kimberley region, presumably associated with the 1993-94 wet season.

BF virus disease was made a notifiable disease in Western Australia in June 1994 as a direct result of

[2] This species is similar to, but distinct from *Coquillettidia linealis*, according to E.N. Marks, the leading Culicid taxonomist in Australia.

the 1993-94 southwest outbreak (Health [Infectious Diseases] Amendment Order 1994, Government Gazette, Western Australia, 24 June 1994). The outbreak was also the first report of a substantial number of cases in the absence of Ross River virus activity anywhere in Australia. Ross River virus is endemic in the Mandurah (Peel) region but only one case of Ross River virus disease was reported from the area during spring-summer (September-February) 1993-94. This is the lowest recorded number of cases for that period in the region since record keeping began in 1984. Environmental conditions and vector mosquito populations in the southwest were unfavorable for Ross River virus transmission during the BF outbreak. In particular, populations of *Ae. camptorhynchus* from October onwards were much smaller than in years when larger numbers of cases of Ross River virus disease were reported (M.D. Lindsay, C.A. Johansen, J.S. Mackenzie, unpublished observations). It is not known whether the BF virus outbreak occurred because BF virus can circulate under conditions that are not suitable for Ross River virus activity or whether extremely low levels of immunity in "virgin" vertebrate host and human populations in the southwest may have enhanced transmission cycles.

Surveillance and epidemiologic studies carried out by this laboratory in the north of Western Australia since 1972 and in the southwest since 1987 have found no convincing evidence of BF virus activity in these regions prior to the events described in this report. No BF virus was isolated from the north of Western Australia before 1989, despite large-scale processing of field-caught mosquitoes over a 17-year period that yielded hundreds of isolates of other arboviruses. Similarly, no BF virus isolate was obtained from more than 400,000 mosquitoes collected throughout the southwest between 1987 and 1992 and processed for virus isolation. Furthermore, an ongoing serosurvey has found no evidence of infection with BF virus in more than 1,000 individuals of 18 vertebrate species collected in the southwest before 1992 (C.A. Johansen, unpublished results). This suggests that the virus responsible for the recent outbreaks was recently introduced to Western Australia. The means of introduction, initially to the northwest and more recently to the southwest of Western Australia, is not known. In view of the activity at Nhulunbuy in the Northern Territory, before the first Western Australia cases in 1992, it is possible that the virus may have been introduced from that region in a viremic human or in livestock. However, little is known about the duration and height of viremia in infected humans or other animals, and it is not known whether person-to-person vector-mediated transmission of Barmah Forest virus can occur. Our laboratory has begun a study to investigate the molecular epidemiology of BF virus, particularly whether the strain of virus responsible for the Western Australia outbreaks was introduced from Eastern Australia or was a local, hitherto undetected, strain.

### Acknowledgments

The authors thank Tony Wright, (Health Department of Western Australia) for assistance with collection of mosquitoes, Margaret Wallace, Rosa Egerton-Warburton, Roger Clifton, and Dora Li for assistance with laboratory processing of mosquito and virus samples, and staff in the Virology Section, State Health Laboratory Service, the Health Department of Western Australia, for carrying out the serologic testing of human sera. This work was funded by a grant from the Health Department of Western Australia.

**Michael D.A. Lindsay, Cheryl A. Johansen, Annette K. Broom, David W. Smith,* and John S. Mackenzie**

Department of Microbiology, The University of Western Australia, Queen Elizabeth II Medical Centre, Nedlands, WA

*State Health Laboratory Services, Queen Elizabeth II Medical Centre, Nedlands, WA

### References

1. Lindsay MD, Smith DW, Johansen C, Mackenzie JS. Barmah Forest virus disease in Western Australia. Western Australian Notifiable Diseases Bulletin 1994; 4: 1-4.
2. Lindsay MD, Johansen C, Smith DW, Wallace MJ, Mackenzie JS. An outbreak of Barmah Forest virus disease in the south-west of Western Australia. Med J Aust. In press.
3. Mackenzie JS, Lindsay MD, Coelen RJ, Hall RA, Broom AK, Smith DW. Arboviruses causing human disease in the Australian zoogeographic region. Arch Virol 1994;136:447-7.
4. Marshall ID, Woodroofe GM, Hirsch S. Viruses recovered from mosquitoes and wildlife serum collected in the Murray Valley of south-eastern Australia, February 1974, during an outbreak of encephalitis. Aust J Exp Biol Med Sci 1982;60:457-70.
5. Vale TG, Carter IW, McPhie KA, James GS, Cloonan MJ. Human arbovirus infections along the south coast of New South Wales. Aust J Exp Biol Med Sci 1986;64:307-9.
6. Boughton CR, Hawkes RA, Naim HM. Illness caused by a Barmah Forest-like virus in New South Wales. Med J Aust 1988;148:146-7.
7. Phillips DA, Murray JR, Aaskov JG, Weimers MA. Clinical and subclinical Barmah Forest virus infection in Queensland. Med J Aust 1990;152:463-6.
8. Hawkes RA, Boughton CR, Naim HM, Myrick BA, Ramsay LG. Barmah Forest virus infections in humans in New South Wales. Med J Aust 1988;146:569-73.

9. Merianos A, Farland AM, Patel M, Currie B, Whelan P, Dentith H, Smith D. A concurrent outbreak of Barmah Forest and Ross River disease in Nhulunbuy, Northern Territory. Comm Dis Intell (Aust) 1992;16:110-1.
10. Broom AK, Mackenzie JS, Lindsay MD, Wright AE. Epidemiology of Murray Valley encephalitis and Kunjin viruses in Western Australia, 1980-89. Arbovirus Research in Australia 1989;5:14-8. CSIRO and Queensland Institute of Medical Research.
11. Lindsay MD, Johansen C, Wright AE, Condon R, D'Ercole M, Smith D, Mackenzie JS. The epidemiology of outbreaks of Ross River virus infection in Western Australia in 1991-1992. Arbovirus Research in Australia 1992;6:72-6. CSIRO and Queensland Institute of Medical Research.
12. Lindsay MD, Broom AK, Wright AE, Johansen CA, Mackenzie JS. Ross River virus isolations from mosquitoes in arid regions of Western Australia: implication of vertical transmission as a means of persistence of the virus. Am J Trop Med Hyg 1993;49:686-96.

### An Outbreak of *Shigella sonnei* Infection Associated with Consumption of Iceberg Lettuce

**To the Editor:** *Shigella sonnei* outbreaks in England and Wales are typically associated with primary schools and nurseries. The mode of transmission is usually from person to person by the fecal-oral route (1). In a June 1994 outbreak of *Sh. sonnei* food poisoning among adults in several countries in North West Europe, the vehicle of infection appeared to be iceberg lettuce (2).

In early June, the Communicable Disease Surveillance Centre (CDSC), Public Health Laboratory Service, received a report of an increase in domestic cases of *Sh. sonnei* infections in Sweden from the Salmnet network—a European international laboratory-based reporting system for human salmonella infections that provides a timely on-line database. In this instance the network was used for shigellosis. Of 100 reported cases of *Sh. sonnei* infection in Sweden, 52 occurred in two outbreaks in mid-May. Many cases seemed to be due to foodborne infection, and iceberg lettuce and peeled frozen prawns were implicated as vehicles of infection. *Sh. sonnei* phage types 2 and 3 alpha were associated with the outbreaks, and phage types 2 and 65 had been isolated from sporadic cases.

A message was sent throughout England and Wales on Epinet (a system for rapid electronic data transfer to all Consultants in Communicable Disease Control [CsCDC] in each District Health Authority, Public Health Laboratories [PHLs] and other agencies involved in infectious disease control) asking for information on possible foodborne *Sh. sonnei* infection to be sent to CDSC and for isolates to be referred to the Laboratory of Enteric Pathogens (LEP) for phage typing.

### Epidemiologic studies

Laboratory reports of *Sh. sonnei* infection received through the routine reporting system at CDSC were scrutinized to determine the age group and sex distributions during weeks 21 to 24.

After the Epinet message, CsCDC and laboratory directors who reported clinical cases for which *Sh. sonnei* was isolated were asked to administer trawling questionnaires to apparently sporadic cases among adults with no recent history of overseas travel. Personal details and history of illness and exposure to particular foods were sought.

Several small outbreaks and clusters were reported during June. CsCDC was asked for results of any analytical epidemiologic studies to CDSC. The results of the national laboratory reporting system are shown in Table 1. Although there were fewer reports in the first 20 weeks of 1994 than in a similar period in 1993, there were more reports in the weeks 21 to 24 and many more reports among adults. The proportion of total reports constituted by those from adults was 66% in weeks 21 to 24 of 1994 compared with 44% with the same period in 1993. The proportion in women in the 2 periods was 42% in 1994 compared with 26% in 1993.

Forty trawling questionnaires were distributed. Almost all case patients (38/40) had eaten various salad items of which the common food was iceberg lettuce. The lettuce had been consumed in restaurants, pubs, and in the homes of the case-patients. The lettuce was purchased from supermarkets, greengrocers' shops, and street markets. In one outbreak in Northampton, 21 (52%) of guests at a party became ill with diarrhea. *Sh. sonnei* was isolated from fecal specimens. Illness was significantly associated with consumption of iceberg lettuce (relative risk 3.68, confidence intervals 1.34 - 10.11, $p = 0.0004$).

The hypothesis that consumption of iceberg lettuce was associated with apparently sporadic *Sh. sonnei* infection in adults was tested by a case-con-
